# Supplementary material for: The Virtual “Enfacement Illusion” on Pain Perception in Patients Suffering from Chronic Migraine: A Study Protocol for a Randomized Controlled Trial
Source: J Clin Med. 2022 Nov 21;11(22):6876. doi: 10.3390/jcm11226876 (PMC9699363; doi:10.3390/jcm11226876)
Supplement: Supplementary file 1 [file jcm-11-06876-s001.zip › jcm-1962536-supplementary.pdf]

STANDARD PROTOCOL ITEMS: RECOMMENDATIONS FOR INTERVENTIONAL TRIALS

SPIRIT 2013 Checklist: Recommended items to address in a clinical trial protocol and related documents.

Section/item	Item No	Description
**Administrative information**		
Title	1	Descriptive title identifying the study design, population, intervention<br>Page 1
Trial registration	2a	Trial identifier and registry name.<br>Trial registration: NCT04904458 on 27 May 2021.<br>Registry name: Clinicaltrials.gov.<br>https://clinicaltrials.gov/ct2/show/NCT04904458<br>Page 14
	2b	World Health Organization Trial Registration Data Set<br>Not applicable
Protocol version	3	Date and version identifier<br>Page 14
Funding	4	Sources and types of financial, material, and other support<br>Page 14
Roles and responsibilities	5a	Names, affiliations of protocol contributors: page 1<br>Roles of protocol contributors: page 13
	5b	Name and contact information for the trial sponsor:<br>Page 13
	5c	Role of study sponsor/funder in study design, data collection, analysis, and interpretation of data; writing of the report.<br>Page 13
	5d	Composition, roles, and responsibilities of the coordinating centre, steering committee, endpoint adjudication committee, data management team, and other individuals or groups overseeing the trial, if applicable (see Item 21a for data monitoring)<br>Not applicable
**Introduction**		
Background and rationale	6a	Description of research question and justification for undertaking the trial, including summary of relevant studies<br>Page 1-3
	6b	Explanation for choice of comparators<br>Page 3
Objectives	7	Specific objectives or hypotheses<br>Page 3

Trial design	8	Description of trial design including type of trial (RCT), and framework (superiority)<br>Page 4

Methods: Participants, interventions, and outcomes

Study setting	9	Description of study setting<br>Page 4
Eligibility criteria	10	Inclusion and exclusion criteria for participants<br>Page 4-5<br>individuals who will perform the intervention<br>Page 7
Interventions	11a	Interventions for each group with sufficient detail to allow replication, including how and when they will be administered<br>Page 7-9<br>Figure 3, 4, 5, 6
	11b	Criteria for discontinuing intervention for a given trial participant<br>page 7, 11
	11c	Strategies to improve adherence to intervention protocols<br>Page 11
	11d	Relevant concomitant care and interventions that are permitted or prohibited during the trial<br>Page 5
Outcomes	12	Primary, secondary, and other outcomes, including the specific measurement variables analysis metric<br>Pages 9-10<br>Table 1
Participant timeline	13	Time schedule of enrolment, interventions (including any run-ins and washouts), assessments, and visits for participants.<br>Page 4<br>Figure 1
Sample size	14	Estimated number of participants needed to achieve study objectives and how it was determined, including clinical and statistical assumptions supporting any sample size calculations<br>Page 10
Recruitment	15	Strategies for achieving adequate participant enrolment to reach target sample size<br>Pages 5

Methods: Assignment of interventions (for controlled trials)

Allocation:

Sequence generation	16a	Method of generating the allocation sequence (eg, computer-generated random numbers), and list of any factors for stratification.<br>Page 6

Allocation concealment mechanism	16b	Mechanism of implementing the allocation sequence (eg, central telephone; sequentially numbered, opaque, sealed envelopes), describing any steps to conceal the sequence until interventions are assigned<br>Page 6
Implementation	16c	Who will generate the allocation sequence, who will enrol participants, and who will assign participants to interventions<br>Page 6
Blinding (masking)	17a	Who will be blinded after assignment to interventions (eg, trial participants, care providers, outcome assessors, data analysts), and how<br>Page 6
	17b	If blinded, circumstances under which unblinding is permissible, and procedure for revealing a participant's allocated intervention during the trial<br>Not applicable
**Methods: Data collection, management, and analysis**		
Data collection methods	18a	Plans for assessment and collection of outcome, baseline, and other trial data;<br>Page 5.<br><br>description of study instruments;<br>Page 5
	18b	Plans to promote participant retention and complete follow-up, including list of any outcome data to be collected for participants who discontinue or deviate from intervention protocols<br>Not applicable
Data management	19	Plans for data entry, coding, security, and storage, including any related processes to promote data quality (eg, double data entry; range checks for data values). Reference to where details of data management procedures can be found, if not in the protocol<br>Pages 10-11
Statistical methods	20a	Statistical methods for analysing primary and secondary outcomes. Reference to where other details of the statistical analysis plan can be found, if not in the protocol<br>Pages 10-11
	20b	Methods for any additional analyses (eg, subgroup and adjusted analyses)<br>Pages 10-11
	20c	Definition of analysis population relating to protocol non-adherence (eg, as randomised analysis), and any statistical methods to handle missing data (eg, multiple imputation)<br>Page 11
**Methods: Monitoring**		
Data monitoring	21a	Composition of data monitoring committee (DMC); Alternatively, an explanation of why a DMC is not needed.<br>Page 11. Not required.

	21b	Description of any interim analyses and stopping guidelines<br>Not applicable.
Harms	22	Plans for collecting, assessing, reporting, and managing solicited and spontaneously reported adverse events and other unintended effects of trial interventions or trial conduct<br>Page 11
Auditing	23	Frequency and procedures for auditing trial conduct, if any, and whether the process will be independent from investigators and the sponsor<br>Not applicable

Ethics and dissemination

Research ethics approval	24	Ethics approval<br>Pages 11, 13
Protocol amendments	25	Plans for communicating important protocol modifications (eg, changes to eligibility criteria, outcomes, analyses) to relevant parties (eg, investigators, REC/IRBs, trial participants, trial registries, journals, regulators)<br>Page 11
Consent or assent	26a	Who will obtain informed consent or assent from potential trial participants or authorised surrogates, and how (see Item 32)<br>Page 5
	26b	Additional consent provisions for collection and use of participant data and biological specimens in ancillary studies, if applicable<br>Not applicable.
Confidentiality	27	How personal information about potential and enrolled participants will be collected, shared, and maintained in order to protect confidentiality before, during, and after the trial<br>Pages 11
Declaration of interests	28	Financial and other competing interests for principal investigators for the overall trial and each study site<br>Page 14
Access to data	29	Statement of who will have access to the final trial dataset, and disclosure of contractual agreements that limit such access for investigators<br>Pages 10-11
Ancillary and post-trial care	30	Provisions, if any, for ancillary and post-trial care, and for compensation to those who suffer harm from trial participation<br>Not applicable, no provisions planned, no major risks associated with study participation
Dissemination policy	31a	Plans for investigators and sponsor to communicate trial results to participants, healthcare professionals, the public, and other relevant groups (eg, via publication, reporting in results databases, or other data sharing arrangements), including any publication restrictions<br>Pages 11

	31b	Authorship eligibility guidelines and any intended use of professional writers<br>All named authors adhere to the authorship guidelines of Trials. All authors have agreed to publication.<br>Page 14
	31c	Plans, if any, for granting public access to the full protocol, participant-level dataset, and statistical code<br>Page 10, 11, 14
**Appendices**		
Informed consent materials	32	Model consent form and other related documentation given to participants and authorised surrogates<br>The consent form and materials are available from the corresponding author on request.
Biological specimens	33	Plans for collection, laboratory evaluation, and storage of biological specimens for genetic or molecular analysis in the current trial and for future use in ancillary studies, if applicable<br>Not applicable.
